# Prosthetic orientation, limb alignment, and soft tissue balance with bi-cruciate stabilized total knee arthroplasty: a comparison between the handheld robot and conventional techniques

**DOI:** 10.1007/s00264-023-05737-6

**Published:** 2023-03-17

**Authors:** Tomoyuki Matsumoto, Naoki Nakano, Shinya Hayashi, Koji Takayama, Toshihisa Maeda, Kazunari Ishida, Yuichi Kuroda, Takehiko Matsushita, Takahiro Niikura, Hirotsugu Muratsu, Ryosuke Kuroda

**Affiliations:** 1 Department of Orthopedic Surgery, Kobe University Graduate School of Medicine, 7-5-1, Kusunoki-cho, Chuo-ku, Kobe, 650-0017 Japan; 2 Department of Orthopaedic Surgery, Anshin Clinic, Kobe, Japan; 3 Department of Orthopedic Surgery, Kobe Kaisei Hospital, Kobe, Japan; 4 Department of Orthopedic Surgery, Hyogo Prefectural Harima-Himeji General Medical Center, Himeji, Japan

**Keywords:** Total knee arthroplasty, Robotic, Conventional, Prosthetic positioning, Limb alignment, Soft tissue balance, Clinical outcomes

## Abstract

**Purpose:**

This study aimed to examine the prosthetic orientations, limb alignment, intraoperative soft tissue balance, and early clinical outcomes associated with the use of the relatively new handheld robot technique compared to those associated with the use of the conventional alignment guide for bi-cruciate stabilized total knee arthroplasty (TKA).

**Methods:**

This retrospective cohort study compared the prosthetic orientation and limb alignment of 35 patients who underwent TKA using robotic assistance (robot group) with those of patients who underwent TKA using a conventional alignment guide (control group). The coronal femoral component alignment (FCA), coronal tibial component alignment (TCA), and the hip-knee-ankle (HKA) angle were compared between groups. Intraoperative soft tissue balance, including the joint component gap and varus/valgus balance assessed by an offset-type tensor, were also compared between groups. One year postoperatively, the clinical outcomes, including the range of motion and 2011 Knee Society Score (KSS), were compared between groups.

**Results:**

The HKA angle and FCA were 0.1° varus and 0.1° varus, respectively, in the robot group and 1.3° varus and 1.3° varus, respectively, in the control group. The difference in the HKA angle and the FCA, but not the TCA, between groups was statistically significant (*p* < 0.05). The intraoperative soft tissue balance showed more stable joint component gaps and varus/valgus balances throughout the range of motion in the robot group than in the control group. Clinical outcomes of the robot group showed superior 2011 KSS subscales compared to those of the control group.

**Conclusion:**

The accuracy of the implantations and stable soft tissue balance in the robot group were superior to those of the control group. The robot group also had superior patient-reported scores for early clinical outcomes.

## Introduction

Achieving optimal limb alignment based on the appropriate prosthetic positioning is recognized as one of the most important measures of good clinical outcomes and long-term survival after total knee arthroplasty (TKA). An alignment error exceeding 3° valgus or varus has been associated with more rapid failure and less satisfactory functional results after TKA [1–3]. Recently, robotic systems have become a promising option for achieving this goal. One systemic review and meta-analysis recently reported 16 studies that compared robotic-arm assisted TKA with manual TKA and revealed that component positioning with the use of a robotic-arm assisted system was more precise in the femur and tibia than those achieved using manual procedures [4].

Recently, handheld robot system (NAVIO Surgical System; Smith & nephew, Memphis, TN, USA) has been introduced, and it is available for TKA. An image-free handheld robotic sculpting system can enable surgeons to plan prosthetic positioning with six degrees of freedom intraoperatively without the need for preoperative planning. Intraoperatively, surgeons can optimize soft tissue balancing, bone cutting, and implant orientation in real time. The accuracy of this system to ensure precise bone cutting has also been reported by a cadaveric study [5]. However, only few studies have investigated the usefulness and clinical outcomes of this system [6, 7]. Furthermore, appropriate soft tissue balance may be achieved because surgeons can easily adjust soft tissue balance in real time when using this system; however, no study has compared intraoperative soft tissue balance achieved with this robot system and that achieved with conventional procedures.

Therefore, in the present study, the prosthetic orientations, limb alignment, intraoperative soft tissue balance, and early clinical outcomes of patients treated using this robotic system were compared to those of patients treated using a conventional alignment guide. In this study, we hypothesized that the robotic system would provide more precise prosthetic positioning, thus leading to more optimal limb alignment, more stable soft tissue balance, and better clinical outcomes compared to those achieved with the conventional procedure used for bi-cruciate stabilized TKA.

## Materials and methods

**Fig. 1:**
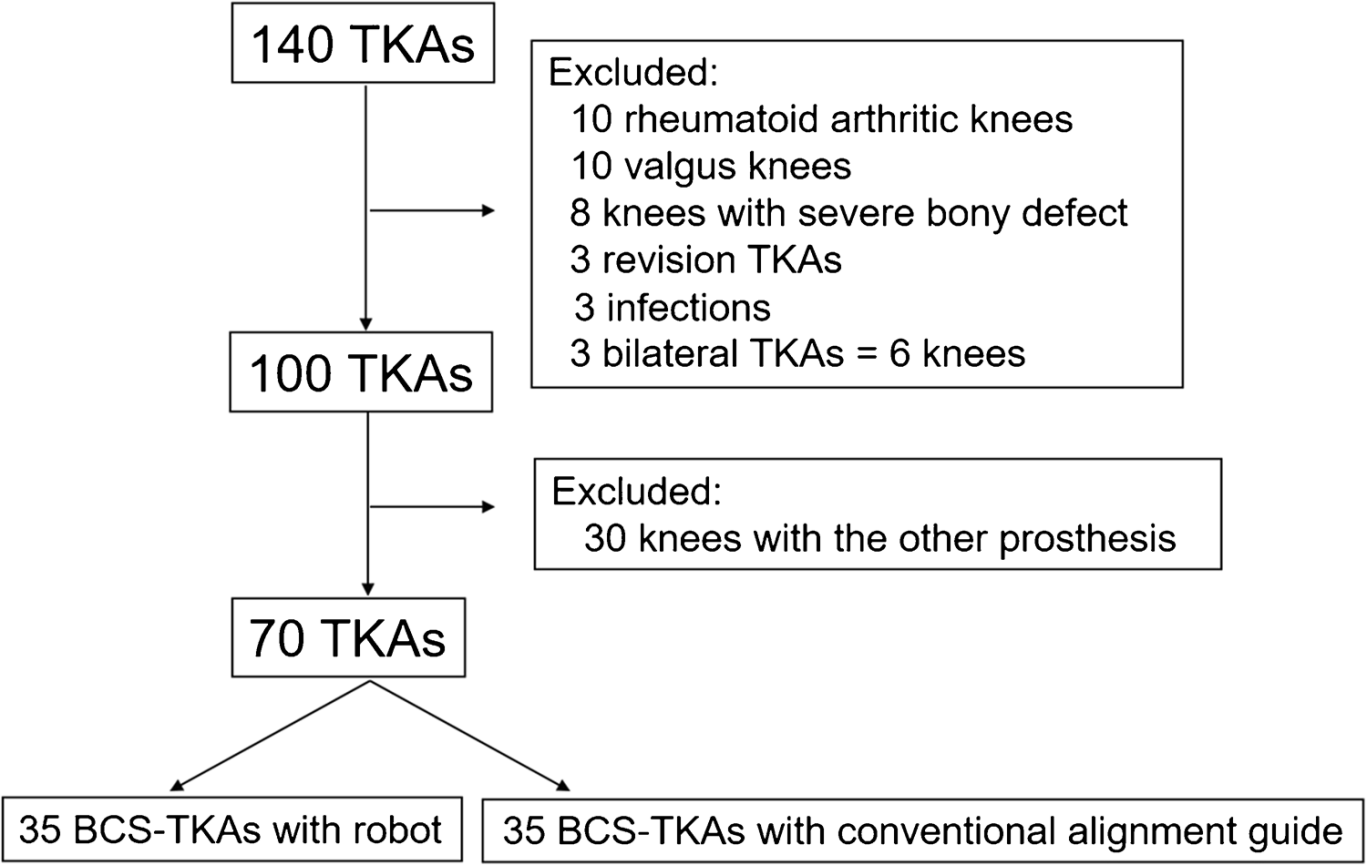
Flowchart of patients undergoing each type of total knee arthroplasty (TKA). BCS-TKA: bi-cruciate stabilized total knee arthroplasty

The hospital ethics committee approved the study protocol, and the patients provided informed consent for participation in the study. The inclusion criteria were substantial pain and loss of function caused by severe osteoarthritis of the knee (Kellgren-Lawrence grade 3–4). To perform a fair assessment and minimize the influences of clinical variables, the exclusion criteria were as follows: knees with valgus deformity, the presence of severe bony defects requiring bone grafts or augmentation, revision TKA, active knee joint infection, and the need for bilateral TKA. From January to December 2020, 35 consecutive patients who met the above criteria were implanted with the *JOURNEY*™ *II* bi-cruciate stabilized (BCS) total knee system (Smith & Nephew, Inc., Memphis, TN, USA) using an image-free handheld robot system (NAVIO Surgical System; Smith & Nephew, Memphis, USA) (robot group) (Fig. 1). The results of this study group were retrospectively compared to those of a control group consisting of 35 subjects with the same type of total knee prostheses implanted using a conventional alignment guide during the study period (control group). Each surgery was performed by the same surgeon (T.M.) with more than ten years of experience performing TKA. The patients’ demographic data included age, sex, diagnosis, body mass index, preoperative deformity, and range of motion (ROM) were similar in both groups (Table 1).

**Table 1:** Patient demographic data

	Robot	Control	*p*value
Patients, no	35	35	N.S
Age (years)*	74.6 (52–88)	74.7 (58–87)	N.S
Sex (% male)	17.1% (6/35)	14.3% (5/35)	N.S
Diagnosis (% osteoarthritis)	100%	100%	N.S
Body mass index**	25.3 ± 4.5	26.5 ± 3.6	N.S
Deformity (varus) (degrees)**#	9.9 ± 4.6	8.0 ± 6.0	N.S
Range of motion			
Extension (°)*	− 12.1 ± 6.9 (− 30–0)	− 9.9 ± 6.7 (− 25–0)	N.S
Flexion (°)*	114.1 ± 15.6 (80–135)	120.6 ± 13.2 (90–135)	N.S

^*^ Data are presented as the mean (range)

** Data are provided as mean ± standard deviation. #: Positive values indicate varus alignment. *N*.*S*. not significant

### Operative procedures: robot group

Preoperative coronal and sagittal long-leg weight-bearing radiographs were obtained and used to select the appropriate femoral and tibial prostheses and to determine the appropriate level and angle of osteotomies in relation to the mechanical axes.

**Fig. 2:**
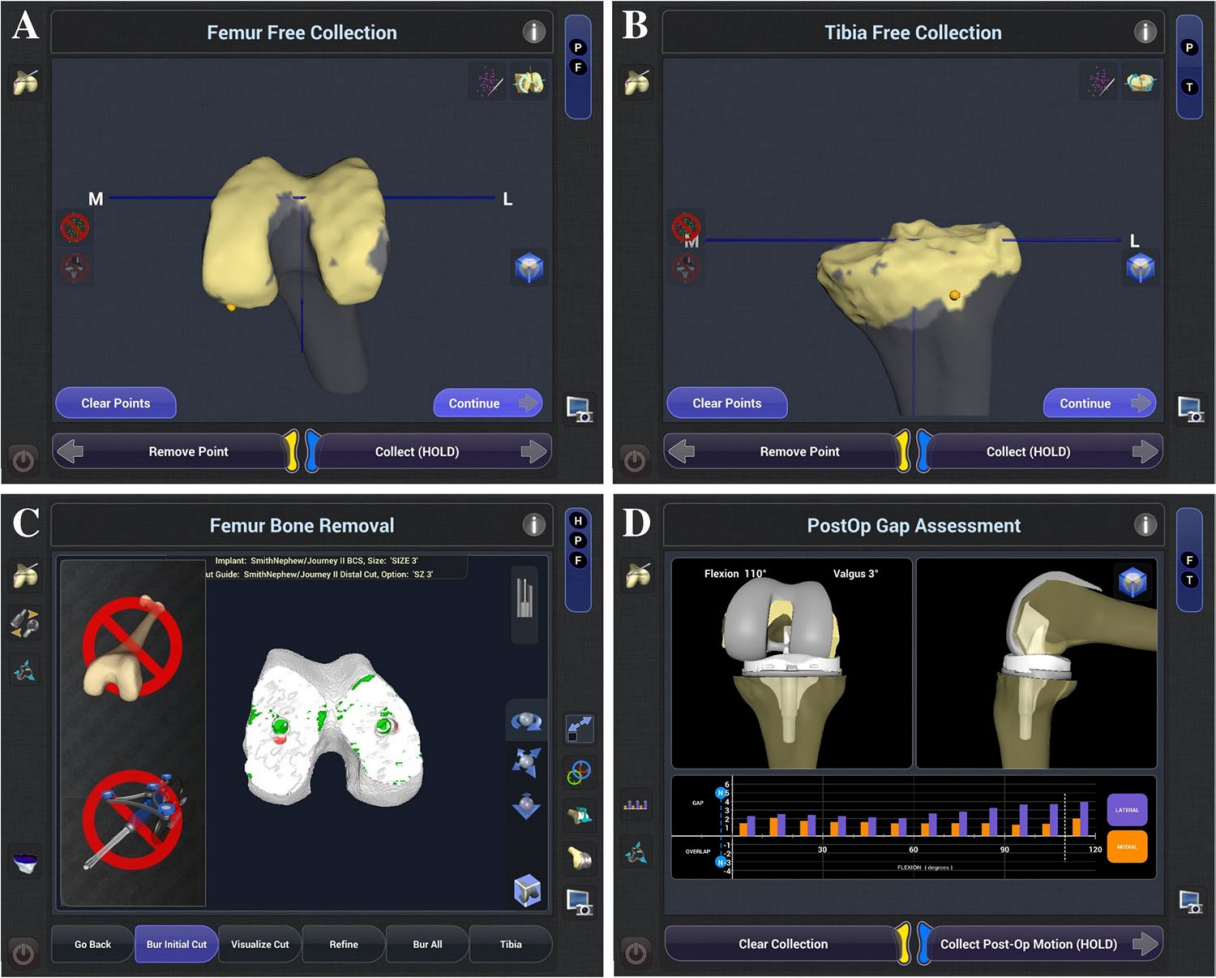
Feature of handheld robot. **A** Bone surface registration. Bone surface of the femur was morphologically registered by morphing and painting technique with the probe. **B** Surface registration of the tibia by morphing technique. Bone surface of the tibia was also morphologically registered by morphing and painting technique with the probe. **C** Distal and anteroposterior femoral cut. Distal femoral shaving was performed, in which targeted cut surface was changed from purple, green, to white color, with a high-speed 5-mm burr. This burr was handheld by the surgeon but controlled by the robot system to avoid shaving the wrong areas. Rotational alignment and anteroposterior position of the femoral component was determined by two peg halls of cutting block, which was created with the burr under robotic control. **D** Soft tissue balance assessment. Intraoperative soft tissue balance was assessed under manual varus/valgus stress throughout the range of motion. Real-time assessment enables surgeons to check the lateral gaps in blue colour and medial gaps in orange colour

The knees were exposed using medial parapatellar arthrotomy along with the use of tourniquets. First, two percutaneous bicortical threaded pins were implanted in the distal femur and in the proximal tibia for the tracking arrays of the robot system. The anterior and posterior cruciate ligaments were sacrificed, and necessary osteophytes were removed before registration with the robot. Before registration could be performed, the patient’s orientation and position during surgery were defined. Mechanical and rotational axes of the limb were determined by establishing the hip, knee, and ankle centres, which were almost the same as those previously reported by navigation systems [8, 9]. The morphology of the femoral and tibial surfaces were determined by morphing and painting the structures of bone surfaces with the probe (Fig. 2A, B). Then, a virtual three-dimensional model of the knee was created using software. Using the monitor, the surgeon could select the implant size, varus/valgus alignment, rotational alignment, and the level to which the bone was to be cut in the coronal, sagittal, and rotational planes. During this series, the height and angle of the distal femoral osteotomy were planned perpendicular to the femoral mechanical axis. A proximal tibial osteotomy was planned perpendicular to the mechanical axis in the coronal plane with 5° of posterior inclination along the sagittal plane. Femoral rotation was planned by adjusting the posterior condylar axis, transepicondylar axis, and Whiteside line.

Thereafter, the soft tissue balance including the medial and lateral gaps throughout the ROM with valgus stress and varus stress was recorded. Based on the parameters including prosthetic positioning and soft tissue balance, the robot software showed a simulation of the soft tissue balance after prosthetic implantation. Therefore, the surgeon could adjust the level to which the bone was to be cut, implant size, and rotation to achieve an appropriate soft tissue balance before cutting the bone. After the planning and simulation, distal femoral shaving was performed with a high-speed 5-mm burr, which was handheld by the surgeon but controlled by the robot system to avoid shaving the wrong areas while the surgeon looked at the mapped area of the femur (Fig. 2C). Anterior and posterior femoral cuts were performed by setting the robot-guided cutting block, which was defined by the rotational alignment of the femoral component. The tibial resection block was set to the optimal position guided by the system, which was similar to the procedure of the navigation systems.

After the trial components and insert were placed, the soft tissue balance could be checked again with valgus stress and varus stress throughout the ROM with a graphical representation of the medial and lateral gaps (Fig. 2D).

### Operative procedures: control group

Surgeries were performed using the conventional measured resection technique with the use of an intramedullary alignment rod for the femur and an extramedullary alignment guide for the tibia. Alignment targets in the control group were the same as those in the robot group, with perpendicular cuts in relation to the femoral and tibial mechanical axes in the coronal plane and 5° of tibial posterior slope.

### Intraoperative measurement of the soft tissue balance

During this study, an offset-type tensor, which enables evaluation of the soft tissue balance throughout the ROM with femoral component placement and patellofemoral joint reduction, was used for the intraoperative measurement of the soft tissue balance [10, 11]. This tensor permits intraoperative reproduction of postoperative alignment of the patellofemoral and tibiofemoral joints; the accuracy of measuring this tensor in patients who underwent TKA has been reported [12, 13]. Before final implantation of the prostheses, the tensor was fixed, and the femoral trial prosthesis and patellofemoral joint were reduced by temporarily suturing the medial parapatellar arthrotomy. Then, the soft tissue balance, including the joint component gap (mm) and varus/valgus ligament balance (°) with the knee at 0°, 10°, 30°, 45°, 60°, 90°, 120°, and 135° of flexion were measured with a distraction force of 40 lb. This distraction force was loaded several times until the joint center gap remained constant. This was performed to reduce the error that can result from creep elongation of the surrounding soft tissues.

### Radiological measurements

The limb alignment and prosthetic position on postoperative long-leg weight-bearing radiographs were assessed. Then, the mechanical axes of the femur and tibia were measured. Using these mechanical axes, three roentgenographic parameters of limb alignment and the component positioning alignment were measured based on the mechanical axis lines for each of 70 TKA procedures: hip-knee-ankle (HKA) angle; coronal femoral component alignment (FCA); and coronal tibial component alignment (TCA). All radiological measurements were performed by one of the authors (N.N.) who was blinded to all the other clinical information.

To determine the intra-observer reliability and inter-observer reliability of the radiographic assessment, two investigators performed all radiographic assessments twice using 20 randomly selected radiographs. The intra-observer reliability and inter-observer reliability of all radiographic measurements were evaluated using intraclass correlation coefficients. The intraclass correlation coefficients for intra-observer reliability and inter-observer reliability were > 0.85 (range, 0.86–0.94) for all measurements. Based on the observed reliability of the results, measurements obtained by a single investigator (N.N.) were used for the analyses.

### Clinical evaluations

Clinical evaluations were performed for each patient one year postoperatively. The ROM and patient-derived 2011 Knee Society Score (KSS) [14], which includes four categories: symptoms, patient satisfaction, patient expectations, and functional activities, such as walking and standing, standard activities, advanced activities, and discretionary activities, were assessed. The objective knee indicators score from the 2011 KSS, determined by the surgeon who was blinded to the group assigned, includes alignment, instability, and joint motion. The patient satisfaction score, patient expectations score, and functional activities score were determined by the patients in the outpatient clinic.

### Statistical analysis

All values assessed were expressed as mean ± standard deviation. The results were statistically analyzed using a statistical software package (Graph Pad Prism software; Graph Pad, San Diego, CA, USA). The non-paired *t* tests were utilized to compare the parameters of the robot and control groups. The number of outliers from the target value within ± 2° varus or valgus were analyzed between groups using the χ^2^ test. *p* < 0.05 was considered statistically significant.

A power analysis was performed using G*Power 3 (Heinrich Heine, University of Dusseldorf, Dusseldorf, Germany) [15]. We calculated that with a sample of 70 patients (35 patients per group), the study would have a power (1-β) of 0.92 to detect a mean difference of 1/2 standard deviation, with a type-I error (α) of 0.05. We also calculated the estimated sample size of 52 patients for the two-group comparison using the same manner as described (1-β = 0.8, effect size = 0.5, α = 0.05).

## Results

### Radiological results

The alignments of the limb and each component are shown in Table 2. The HKA angle was 0.1 ± 1.1° varus in the robot group and 1.3 ± 2.2° varus in the control group. The FCA and TCA were 0.1 ± 1.1° varus and 0.1 ± 1.0° valgus in the robot group, respectively, and 1.3 ± 1.9° varus and 0.0 ± 1.4° varus in the control group, respectively (Table 2). There were significant differences in the HKA angle and the FCA, but not in the TCA, between groups.

**Table 2:** Postoperative radiological parameters

	Robot	Control	*p*value
HKA angle (°)	0.1 ± 1.5 varus (4.0 valgus – 3.0 varus)	1.3 ± 2.2 varus (4.0 valgus – 5.0 varus)	< 0.001
FCA (°)	0.1 ± 1.1 varus (2.0 valgus – 3.0 varus)	1.3 ± 1.9 varus (4.0 valgus – 5.0 varus)	0.002
TCA (°)	0.1 ± 1.0 valgus (4.0 valgus – 3.0 varus)	0.0 ± 1.4 varus (4.0 valgus – 3.0 valgus)	N.S

Data are presented as the mean ± standard deviation

*HKA* hip-knee-ankle, *FCA* femoral component alignment, *TCA* tibial component alignment, *N*.*S*. not significant

**Fig. 3:**
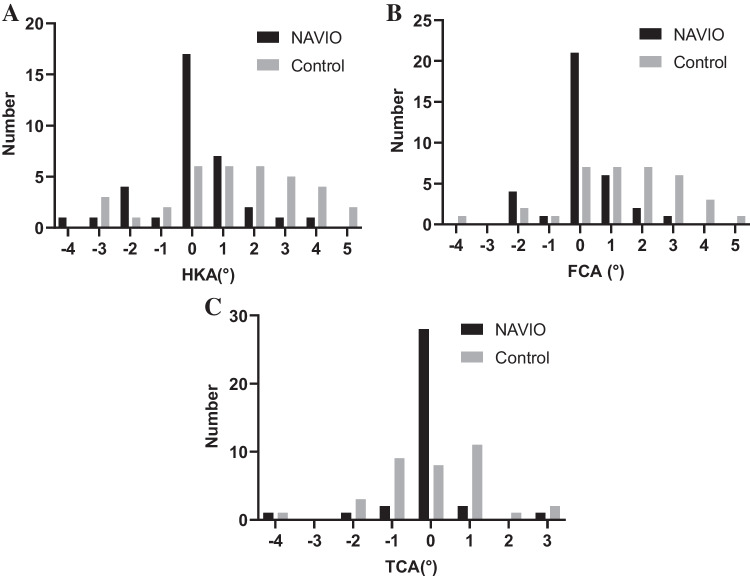
Histograms of radiological parameters. **A** Hip-knee-ankle (HKA) angle. The HKA angle within 2° varus/valgus was obtained for 31 cases (11.4% outliers) in the robot group and 21 cases (40.0% outliers) in the control group. **B** Femoral component alignment (FCA). The FCA within 2° varus/valgus was obtained for 34 cases (2.9% outliers) in the robot group and 26 cases (25.7% outliers) in the control group. **C** Tibial component alignment (TCA). The TCA within 2° varus/valgus was obtained for 33 cases (5.7% outliers) in the robot group and 32 cases (8.6% outliers) in the control group

The HKA angle within 2° varus or valgus was obtained for 31 cases in the robot group (outliers: 11.4%) and 21 cases in the control group (outliers: 40.0%). The FCA and the TCA within 2° varus or valgus were obtained for 34 cases (outliers: 2.9%) and 33 cases (outliers: 5.7%) in the robot group, and for 26 cases (outliers: 25.7%) and 32 cases (outliers: 8.6%) in the control group, respectively. The HKA angle and the FCA, but not the TCA, differed significantly between groups (*p* = 0.01 for both). Histograms of these data are provided in Fig. 3A, B, and C.

### Intraoperative soft tissue balance

**Fig. 4:**
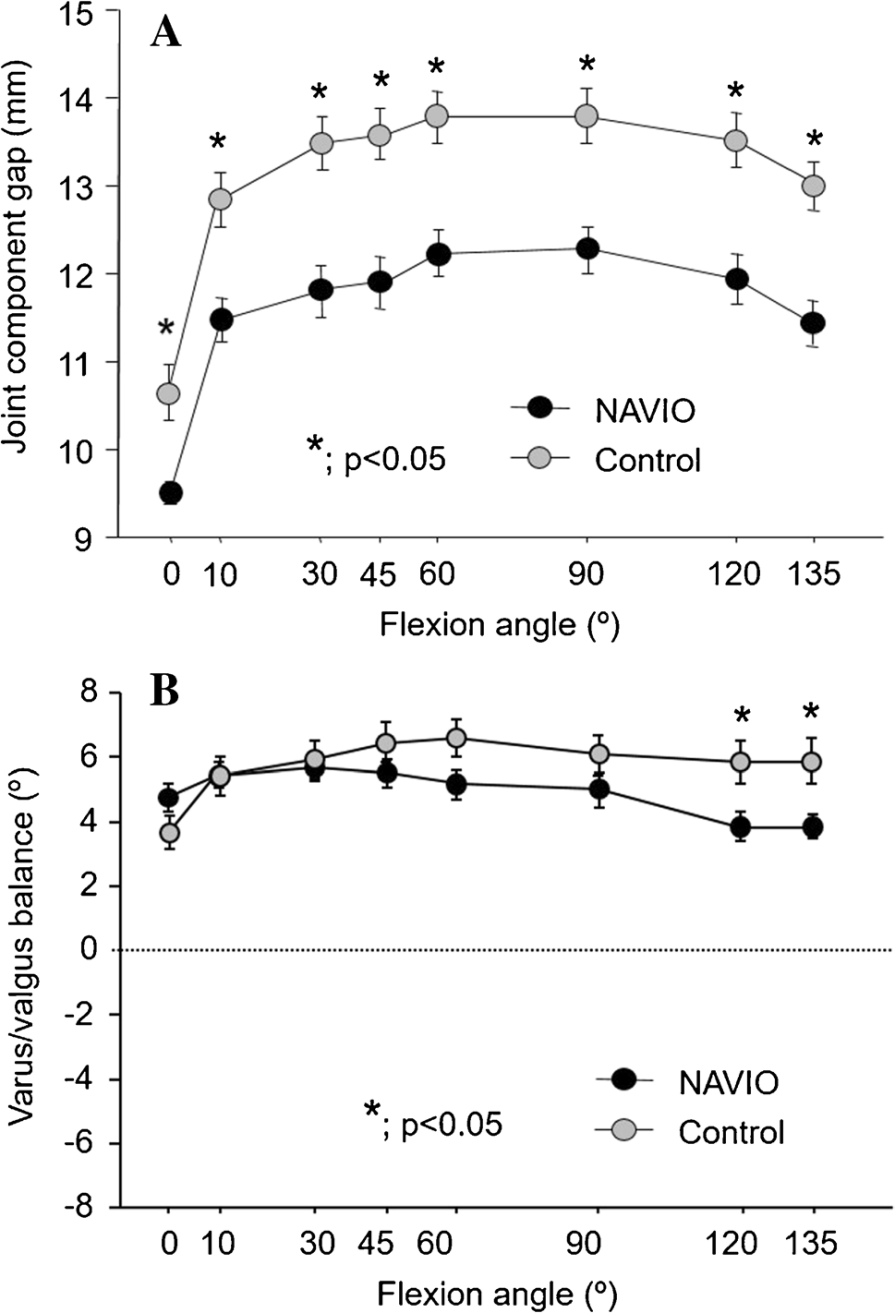
Intraoperative soft tissue balance. **A** Joint component gap. The joint component gaps in the participants in the robot group were significantly smaller throughout the range of motion than those in the control group. *p < 0.05. **B** Varus/valgus ligament balance. The varus/valgus ligament balance in the participants exhibited slight lateral laxity throughout the range of motion. The value in the robot group were significantly smaller at 120 and 135° of flexion than those in the control group. *p < 0.05

The mean joint component gaps of the knee are shown in Table 3. The joint component gaps throughout the ROM were significantly smaller in the robot group than in the control group (Fig. 4A).

**Table 3:** Intraoperative soft tissue balance

	Robot	Control	*p*value
Joint component gap (mm)			
0	9.5 ± 0.7	10.7 ± 1.7	0.01
10	11.5 ± 1.3	12.9 ± 1.6	< 0.001
30	11.8 ± 1.6	13.6 ± 1.7	< 0.001
45	11.9 ± 1.6	13.5 ± 1.7	< 0.001
60	12.2 ± 1.5	13.9 ± 1.6	< 0.001
90	12.3 ± 1.4	13.6 ± 1.8	< 0.001
120	11.9 ± 1.5	14.0 ± 1.9	< 0.001
135	11.4 ± 1.4	13.8 ± 1.9	< 0.001
Varus/valgus balance (°)			
0	4.7 ± 2.2	3.4 ± 2.7	N.S
10	5.4 ± 2.0	5.2 ± 3.1	N.S
30	5.7 ± 2.3	5.7 ± 3.2	N.S
45	5.5 ± 2.4	6.2 ± 3.4	N.S
60	5.1 ± 2.4	6.3 ± 3.3	N.S
90	5.0 ± 2.4	5.8 ± 3.3	N.S
120	3.9 ± 2.5	5.6 ± 3.7	0.038
135	3.9 ± 2.0	5.6 ± 3.8	0.033

Data are presented as the mean ± standard deviation, *N*.*S*. not significant

The mean varus/valgus ligament balance (positive value indicates varus balance) of the knee is shown in Table 3. The varus/valgus ligament balance in participants exhibited slight lateral laxity throughout the ROM. During deep flexion, the varus balances in the robot group were significantly smaller than those in the control group (Fig. 4B).

### Insert thickness

Selected insert thicknesses were 9.7 ± 0.9 mm in the robot group and 9.9 ± 1.1 mm in the control group. However, the difference was not statistically significant.

### Clinical results

The average postoperative ROMs was not no significantly different between groups (Table 4). The postoperative 2011 KSS for the objective indicators, patient satisfaction, and functional activity score, but not for patient expectations, were significantly better in the robot group than in the control group (*p* < 0.05) (Table 4).

**Table 4:** Postoperative clinical outcomes

	Robot	Control	*p*value
ROM			
Extension (°)	− 2.9 ± 4.2 (− 15–0)	− 2.1 ± 3.2 (− 10–0)	N.S
Flexion (°)	121.3 ± 13.1 (80–140)	125.1 ± 13.2 (110 –140)	N.S
2011 KSS			
Objective knee indicators (100)	94.5 ± 3.7 (87–100)	91.0 ± 6.2 (75–100)	0.013
Patient satisfaction (40)	29.7 ± 7.2 (12–40)	24.7 ± 7.0 (6–36)	0.009
Patient expectations (15)	11.8 ± 2.3 (8–15)	10.4 ± 3.2 (5–15)	N.S
Functional activities (100)	77.2 ± 13.6 (52–98)	63.6 ± 18.1 (33–93)	0.002

Data are presented as the mean ± standard deviation

*ROM*: range of motion; *KSS*: Knee Society Score; *N.S*. not significant

## Discussion

The most important finding of the present study was that the novel image-free, handheld, robot-sculpting system achieved more precise limb alignment/prosthetic positioning, and more stable intraoperative soft tissue balance with bi-cruciate stabilized TKA compared with the conventional alignment guide system. Furthermore, superior clinical outcomes according to the 2011 KSS were noted in the robot group one year postoperatively. These findings as well as implant longevity must be confirmed by studies with a longer follow-up period, however.

During the present study, the novel robot system was able to achieve neutral implantation in the FCA (0.1 ± 1.1° varus) and reduce outliers from the target neutral values within 2° varus or valgus (outliers: 2.9%); conversely, the conventional system achieved 1.3 ± 1.9° varus with 25.7% outliers in the control group. The accurate femoral positioning with this robotic system also resulted in a more accurate neutral HKA angle (0.1 ± 1.1° varus) with fewer outliers (11.4% outliers) as compared with those obtained by the conventional alignment guide system (HKA angle: 1.3 ± 2.2° varus, 40.0% outliers). Similarly, Bollars et al. reported that the NAVIO system and conventional technique were associated with 14% and 19% outliers for coronal FCA, respectively, and with 0% and 8% outliers for coronal TCA, respectively. Furthermore, during the assessment of the mechanical axis, 6% and 18% outliers were reported for the robot group and control group, respectively [6]. Collins et al. reported 6.7% outliers from neutral within 3° varus or valgus with the use of the same robotic system [7]. Therefore, similar to another robotic system, the hand-held NAVIO robot-assisted system could provide accurate limb alignment and prosthetic positioning.

The usefulness of this robotic system should be compared with that of other navigation systems. Several meta-analyses have already concluded that compared with conventional alignment guides, navigation systems achieve superior restoration of the mechanical axis of the lower limb with more precise component orientation [16–19]. During a previous study, the use of a navigation system for TKA by the same surgeon as in the present study achieved 7% outliers for the FCA and 7% outliers for the TCA [20]. Compared to the accuracy in that previous study, the accuracy in the present study using the NAVIO robot system was superior. The advantage of this robotic system over the navigation system may be proven after a careful investigation and long-term follow-up.

Regarding the assessment of the intraoperative soft tissue balance, more stable joint component gap and varus/valgus balance were achieved in the robot group than in the control group. These differences may be attributable to the real-time adjustment of the intraoperative soft tissue balance when using this robotic system. In particular, the component gaps throughout the ROM in the robot group were more than 1 mm smaller than those in the control group, suggesting that the robotic system could avoid excessive bone cutting with real-time simulation of the final soft tissue balance adjustment. As a result, the selected insert thickness was tended to be thinner in the robot group than in the control group (average: 9.7 mm vs. 9.9 mm). With the use of sensor-guided technology, 96.7% of the patients with a balanced knee and 82.1% of the patients with an un-balanced knee were satisfied after TKA [21]. Similar to this study, a real-time assessment with this robot system might contribute to stable soft tissue balance in the robot group compared with the control group.

The strength of this study was that the surgeries were performed with the same prosthesis by the same surgeon in the robot group and control group. However, there were several limitations to this study. First, the assessments were performed for a small patient population and excluded valgus deformities. Additionally, this was a retrospective cohort stud; it was not a matched case-controlled study. However, the patients’ demographic data including age, sex, diagnosis, body mass index, preoperative deformity, and ROM showed no statistical differences between groups. Furthermore, the clinical outcomes were assessed as early as one year after surgery; therefore, this may not have resulted in true clinical relevancy.

In conclusion, compared with the conventional technique, the NAVIO handheld robot system achieved more precise restoration of the limb alignment and component orientations and more stable soft tissue balance throughout the ROM with comparable clinical outcomes. This preliminary observation should be investigated in the future to verify the usefulness of this system.
